# Functional characterization of genetic variants affecting the intracellular domains of ATP-binding cassette transporter A1 (ABCA1)

**DOI:** 10.1016/j.jlr.2025.100854

**Published:** 2025-07-03

**Authors:** Marianne Teigen, Åsa Schawlann Ølnes, Katrine Bjune, Martin Prøven Bogsrud, Thea Bismo Strøm

**Affiliations:** 1 Unit for Cardiac and Cardiovascular Genetics, Department of Medical Genetics, Oslo University Hospital, Oslo, Norway; 2 Faculty of Medicine, University of Oslo, Oslo, Norway; 3 Department of Pharmaceuticals and Biomedicine, Norwegian Directorate of Health, Oslo, Norway

**Keywords:** HEK293 cells, Tangier disease, cholesterol efflux, HDL, lipid metabolism, lipoproteins

## Abstract

The ATP-binding cassette transporter A1 (ABCA1) effluxes cellular cholesterol and phospholipids to extracellular acceptors, mainly apolipoprotein A1, generating high-density lipoprotein (HDL) particles. This is the first step in the anti-atherosclerotic process of transporting excess cholesterol from non-hepatic tissues to the liver. Loss-of-function variants in *ABCA1* lead to reduced HDL cholesterol levels in plasma, thus possibly diminishing the atheroprotective effect of HDL. More than 250 missense variants have been reported in the *ABCA1* gene, most of which remain to be functionally characterized. In this study, we have characterized 74 variants affecting the intracellular domains of ABCA1 by assessing cholesterol efflux activity and cell surface localization of the protein, thereby shifting the pathogenicity classification of 10 variants from class 3 (uncertain significance) to class 4 (likely pathogenic) or class 2 (likely benign). Consequently, functional characterization contributes to a better understanding of the molecular basis of the pathogenicity of genetic variants in *ABCA1*, which could also clarify the mechanism of action of the protein.

Dyslipidemia stands out as a critical risk factor for atherosclerosis, the leading cause of coronary artery disease (CAD), which is a major contributor to global mortality (1). Effective cholesterol management is essential in combating this condition. High-density lipoproteins (HDLs) play a vital role by transporting cholesterol from peripheral tissues, including atherosclerotic plaques, back to the liver for excretion via bile and feces (2). This process, known as reverse cholesterol transport, is initiated when apolipoprotein A1 (ApoA1) interacts with ATP-binding cassette transporter A1 (ABCA1), commencing the transfer of cellular cholesterol and phospholipids to ApoA1, forming nascent HDL (3). Defects in this initial step lead to significantly reduced plasma HDL cholesterol (HDL-C) levels, as evidenced in familial HDL deficiency (FHD) and the more severe recessive disorder Tangier disease (4, 5). Tangier disease results from either homozygosity or compound heterozygosity for pathogenic variants in *ABCA1* and is characterized by extremely low HDL-C levels, often below 5 mg/dl (0.13 mmol/l). This condition also involves the accumulation of cholesterol in peripheral tissues and an increased risk of premature CAD (6). In contrast, heterozygosity for pathogenic variants in *ABCA1* or *APOA1* typically results in a milder phenotype associated with FHD (7). However, individuals with FHD still have an increased risk of premature CAD due to significantly reduced HDL-C levels (4, 7, 8).

The ABCA1 protein is a plasma membrane transporter in the ABC superfamily. It consists of two large extracellular domains (ECDs) and two transmembrane domains (TMDs) as well as two intracellular nucleotide-binding domains (NBD1 and NBD2) followed by regulatory domains (R1 and R2, respectively) (9). Moreover, both NBD domains contain three well-conserved motifs reported to be important for ATP-binding, namely the Walker A motif (residues 926–942 and 1939–1955), Walker B motif (residues 1056–1065 and 2068–2077) and the signature motif (residues 1033–1040 and 2045–2052) (10, 11). Although the precise mechanism of ABCA1 remains largely unclear, recent research indicates that the extracellular interaction between ABCA1 and ApoA1, coupled with ABCA1-dependent lipid translocation, is important for effective cholesterol efflux (12, 13, 14, 15, 16). Furthermore, the ATP-binding and -hydrolysis activity of the intracellular NBDs is essential for ABCA1 functionality (17). ATP-binding induces conformational changes in ABCA1, bringing the NBDs and TMDs closer together, which in turn induces conformational changes in the interface between the TMDs and ECDs. This facilitates lipid transfer from the plasma membrane to a hydrophobic tunnel in the ECDs, enabling subsequent lipid transfer to ApoA1 or HDL. ATP-hydrolysis then reverses these conformational changes, returning ABCA1 to its pre-lipid translocation state (16, 18, 19). The importance of the ATPase activity is demonstrated by the variants p.K939M and p.K1952M in the Walker A motif of NBD1 and NBD2, respectively. Both variants fully impede the ABCA1 ATPase activity and result in ABCA1 proteins with no lipid efflux activity, no ApoA1-binding, and no lipid translocation (17, 20, 21, 22).

The Human Gene Mutation Database (HGMD) currently lists more than 350 variants in the *ABCA1* gene, many of which have not yet been functionally characterized. We previously published a study that focused on the functional characterization of missense variants affecting the extracellular domains of ABCA1 (23). In the present study, we extend this analysis to 74 missense variants impacting the intracellular domains of ABCA1, evaluating cell surface localization and cholesterol efflux using a fluorescence-based cholesterol efflux assay. These functional characterizations facilitate the evaluations of pathogenicity in clinical variant assessment in accordance with the American College of Medical Genetics and Genomics and the Association for Molecular Pathology (ACMG/AMP) guidelines, thereby enhancing the diagnostic accuracy of genetic testing. Moreover, this work contributes to our understanding of the ABCA1 mechanism of action, potentially unveiling new therapeutic targets addressing dyslipidemia.

## Materials and Methods

### Nomenclature

Transcript NM_005502.4 of *ABCA1* was used for numbering nucleotides and codons, setting the ATG start codon as codon number 1, and A of the start codon as nucleotide number 1. Variants at the protein level are in this article referred to with the prefix “p.” for clarification, except in figures.

### Variant inclusion

This study includes all previously reported but not functionally characterized *ABCA1* missense variants located in the sequence encoding the intracellular regions of ABCA1. These regions include the two NBDs, the R domains, the intracellular coupling helices (IHs) 1, 2, 3, and 4, the C-terminus, and the regions connecting these and the TMDs, as described by Qian *et al.* (9). This constituted in total 70 variants reported in HGMD (www.hgmd.cf.ac.uk) per June 2022, two novel variants identified in patients with hypoalphalipoproteinemia at the Unit for Cardiac and Cardiovascular Genetics, Oslo University Hospital, Norway, and two variants reported in the literature, but not yet indexed in HGMD (24). All variants included are described in supplemental Table S1.

### The ACMG/AMP guidelines and variant classification

The ACMG/AMP guidelines are considered the global standard for interpreting pathogenicity of genetic variants (25). These guidelines use a five-tier terminology system to categorize variants according to pathogenicity, namely as benign (class 1), likely benign (class 2), uncertain significance (class 3), likely pathogenic (class 4), or pathogenic (class 5), and consist of criteria providing evidence for pathogenicity evaluation, weighed as supporting, moderate, strong, very strong, or stand-alone (26).

To evaluate the ACMG/AMP class of *ABCA1* variants in this study, the filtering allele frequency cutoffs used for criteria BA1 and BS1 were set at ≥ 0.005 and ≥ 0.002, respectively, while the highest allele frequency cutoff for the PM2 criterion was ≤ 0.0002 (27). The phenotype criterion PP4 was applied as supporting evidence for variants observed in an individual with HDL-C < 0.5 mmol/l, moderate if HDL-C < 0.3 mmol/l, or strong if HDL-C < 0.1 mmol/l or Tangier disease.

### Cell culture

Human embryonic kidney 293 (HEK293) cells (European Collection of Authenticated Cell Cultures, Wiltshire, United Kingdom), with minimal endogenous *ABCA1* expression, were cultured in HyClone Minimum Essential Medium (MEM; GE Healthcare Life Sciences, Pittsburg, PA) supplemented with 10% fetal bovine serum, 2 mM L-glutamine (Sigma-Aldrich, St Louis, MO), 50 U/ml penicillin, 50 μg/ml streptomycin (GE Healthcare Life Sciences) and non-essential amino acids (Biowest, Nuaillé, France). Cells were harvested by scraping in lysis buffer (1% Triton X-100 (Sigma-Aldrich), 150 mM NaCl and 10 mM Tris/HCl (pH 7.4)) containing Complete™ Protease Inhibitor Cocktail (Roche Diagnostics GmbH, Mannheim, Germany).

### Plasmids and transfections

The plasmid pcDNA3.1-WT-*ABCA1*-V5/his (WT ABCA1) (23) was used as template to construct the different variants using QuickChange II XL Mutagenesis Kit (Agilent Technologies, Santa Clara, CA) or Q5® Site-Directed Mutagenesis Kit (New England Biolabs, Ipswich, MA), according to the manufacturer’s instructions. The well documented loss-of-function variants p.W590S, p.K939M, p.Y1767D and p.K1952M were used as controls (23). The transgene sequences were validated as previously described (23). Mutagenesis primers are given in supplemental Table S2.

For transient transfection, FuGENE HD (Roche Diagnostics GmbH) was used according to the manufacturer’s instructions, transfecting the cells for 24 hours (h) in a 3:1 ratio to 200 ng/cm^2^ DNA. The transfection efficiency was validated by quantitative PCR to ensure equal expression among the *ABCA1* variants (supplemental Fig. S1).

### Isolation of HDL

HDL (ρ = 1.080–1.21 g/ml) was isolated by ultracentrifugation of serum from healthy blood donors as previously described (23). The lipid and protein concentrations of the HDL fraction were measured at the Department of Medical Biochemistry, Oslo University Hospital, Norway, using standard methods. An ApoA1 concentration of > 5.0 g/l was accepted.

### Cholesterol efflux assay

Cholesterol efflux from HEK293 cells transiently transfected with *ABCA1* variants was assessed as previously described (23). Briefly, the cells were loaded for 1 h at 37°C with BODIPY-cholesterol (Cayman Chemicals, Ann Arbor, MI) in MEM containing 0.0045 mM BODIPY-cholesterol, 0.018 mM cholesterol, 5 mM HEPES, 2.5 mM methyl-β-cyclodextrin, 2% fatty acid free bovine serum albumin, 1% fetal bovine serum (Sigma-Aldrich) and 1 μg/ml avasimibe (Selleck Chemicals LLC, Houston, TX), equilibrated in MEM without phenol red (Gibco Life Technologies, Paisley, UK) supplemented with 1 μg/ml avasimibe for 12–16 h, before incubation in acceptor medium containing 100 μg/ml HDL at 37°C for 4 h. The BODIPY signal in triplicates of 100 μl medium and 10 μg lysate was measured using a Synergy H1 Plate Reader (BioTek, Winooski, VT) with an excitation and emission wavelength of 480 nm and 508 nm, respectively. The formula ([medium signal/(medium signal + lysate signal)]) was used to calculate the cholesterol efflux. HDL-nonspecific cholesterol leakage from cells was adjusted for by subtracting cholesterol efflux to medium without acceptor. ABCA1-nonspecific cholesterol efflux was adjusted for by subtracting efflux from mock-transfected cells. The well-documented loss-of-function variant p.W590S displayed the highest cholesterol efflux activity among several loss-of-function controls in previous experiments and was therefore used as a cutoff for loss-of-function characterization (23).

### Western blot analyses

Cell lysates were examined by Western blot analyses as previously described (23). ABCA1-V5 protein was quantified using an HRP-conjugated anti-V5 antibody from Invitrogen (R961-25; Carlsbad, CA). β-actin was used as a loading control and was quantified by an anti-β-actin antibody from Abcam (ab213262; Cambridge, UK).

### Cell surface biotinylation

Cell surface ABCA1 was quantified in biotinylation assays, as previously described (23). Briefly, transiently transfected HEK293 cells were incubated at 4°C for 30 min with 1 mg/ml EZ-Link Sulfo-NHS-LC-Biotin (Thermo Scientific, Rockford, IL) and then quenched at 4°C for 30 min with 100 mM glycine, harvested and lysed by incubation at −80°C for 30 min, followed by centrifugation to remove cell debris. The lysate was investigated by Western blot analysis after immunoprecipitation using Dynabeads™ MyOne™ Streptavidin T1 (Invitrogen).

### Statistical analyses

Data are presented as mean (± standard deviations) of four independent experiments unless otherwise stated, and individual data points are shown in Supplemental Data S2. *P* values were calculated with a two-sample and two-tailed (unless stated otherwise) *t-*test assuming equal or unequal variance, depending on results from an F-test checking for equal variances, using Stata version 18.0 (StataCorp LLC, College Station, TX).

## Results

### Cholesterol efflux activity

**Fig. 1:**
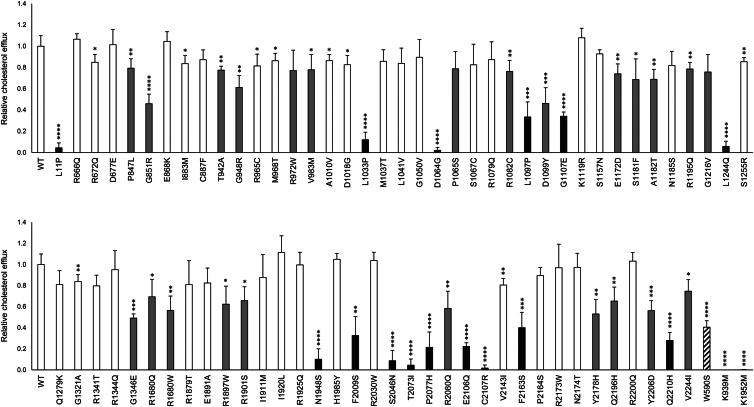
Cholesterol efflux activity of *ABCA1* variants. Relative cholesterol efflux from HEK293 cells transiently transfected with 74 *ABCA1* variants and three known loss-of-function control variants (striped columns) from four independent experiments, normalized to WT ABCA1 (WT). Variants were assessed to be functionally normal (efflux > 80% of WT, white columns), loss-of-function (efflux < 41% of WT, black columns), or of uncertain significance (efflux 41%–80% of WT, gray columns). Error bars represent 1 SD. ∗*P* < 0.05, ∗∗*P* < 0.01, ∗∗∗*P* < 0.001, ∗∗∗∗*P* < 0.0001, *t-*test versus WT ABCA1.

The cholesterol efflux activity of 74 *ABCA1* variants, along with three loss-of-function control variants (p.W590S, p.K939M and p.K1952M), was assessed in transiently transfected HEK293 cells (Fig. 1). Fifteen variants (p.L11P, p.L1033P, p.D1064G, p.L1097P, p.G1107E, p.L1244Q, p.N1948S, p.F2009S, p.S2046N, p.T2073I, p.P2077H, p.E2106Q, p.C2107R, p.F2163S, and p.Q2210H) presented cholesterol efflux below 41% of WT ABCA1 (corresponding to the efflux activity of the loss-of-function control variant p.W590S), thereby characterizing them as loss-of-function variants.

Further, 35 variants exhibited cholesterol efflux activities exceeding 80% of WT ABCA1 and were characterized as functionally normal. This threshold correlates with the average cholesterol efflux observed of the two variants p.I883M (84 ± 8%) and p.V2244I (75 ± 11%). The allele frequency of p.I883M is reported at 67% in the East Asian population according to the Genome Aggregation Database (gnomAD v4.1.0). Moreover, the multiple sequence alignment (28) (supplemental Fig. S2) containing reference sequences from 28 different species reveals that six of the vertebrate species listed have isoleucine (I) in p.2244, indicating that this variant does not impact ABCA1 functionality. An equivalent efflux threshold was set for variants affecting the extracellular domains, supporting the characterization of the 35 functionally normal variants herein (23). The remaining 24 variants, with cholesterol efflux activities ranging from 41% to 80% of WT ABCA1, were characterized as variants of uncertain significance (Fig. 1).

In alignment with our previous findings (23), no consistent correlation between cholesterol efflux activity and the total amount of overexpressed ABCA1 in the cell lysates could be established (supplemental Fig. S3).

### Amount of cell surface ABCA1

**Fig. 2:**
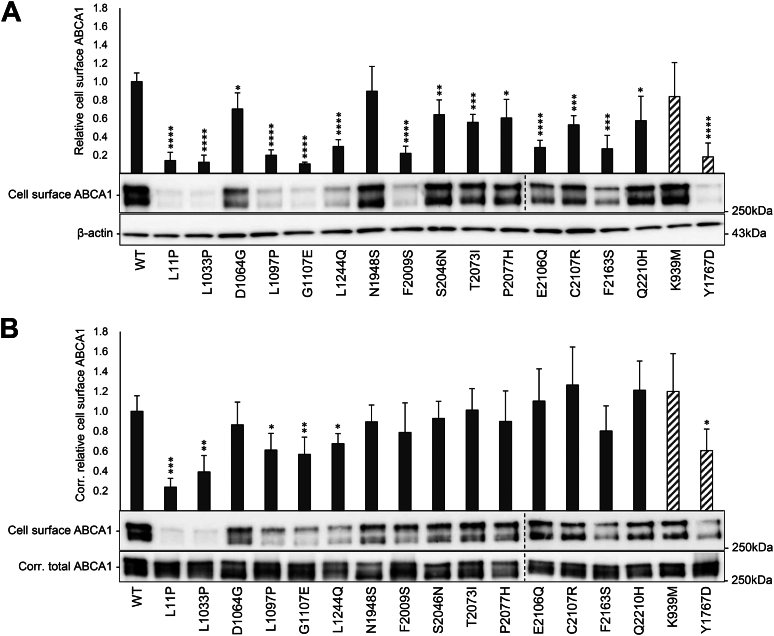
Cell surface expression of the 15 loss-of-function variants. Relative amounts of cell surface ABCA1 were evaluated in HEK293 cells transiently transfected with 15 loss-of-function variants (black columns) and two control variants (striped columns), normalized to WT ABCA1 (WT) in four independent experiments. Error bars represent 1 SD. ∗*P* < 0.05, ∗∗*P* < 0.01, ∗∗∗*P* < 0.001, ∗∗∗∗*P* < 0.0001, *t-*test versus WT ABCA1. One representative Western blot showing cell surface ABCA1 detected using a V5-HRP antibody is presented. Dotted lines designate the merger of blots. A: Total cell surface ABCA1. β-actin in cell lysates was used as a loading control. B: Cell surface ABCA1 corrected (corr.) for amounts of ABCA1 in total lysates. ABCA1 in corrected amounts of cell lysates (corr. total ABCA1) was used as a loading control.

The cell surface localization of ABCA1 is important for its functionality, and several pathogenic *ABCA1* variants have been demonstrated to reduce cell surface ABCA1 protein (23, 29). We therefore assessed the amount of cell surface ABCA1 in HEK293 cells transiently transfected with the 15 identified loss-of-function variants, including two control variants previously demonstrated to have unaffected (p.K939M) and reduced (p.Y1767D) amounts of cell surface ABCA1 (23) (Fig. 2). Whereas p.N1948S was comparable to WT ABCA1, the 14 other loss-of-function variants had reduced amounts of cell surface ABCA1 (Fig. 2A), eight of which (p.L11P, p.L1033P, p.L1097P, p.G1107E, p.L1244Q, p.F2009S, p.E2106Q, and p.F2163S) presented less than 50% of that of WT ABCA1, likely explaining their effect on cholesterol efflux. Whereas both degradation and transport deficiency will result in reduced cell surface levels of proteins, identifying the main mechanism has implications for potential therapeutic strategies. We therefore corrected the cell surface levels of each variant to the total ABCA1 levels in each sample to distinguish transport deficiency from an increased degradation rate (Fig. 2B). Only five of the loss-of-function variants (p.L11P, p.L1033P, p.L1097P, p.G1107E, and p.L1244Q) presented transport deficiency with a continued reduced amount of cell surface ABCA1, two of which (p.L11P and p.L1033P) upheld less than 50%. This indicates that most of the variants are susceptible to an increased degradation rate, resulting in a reduced level of protein at the cell surface.

### Functional rescue of *ABCA1* loss-of-function variants by a chemical chaperone

**Fig. 3:**
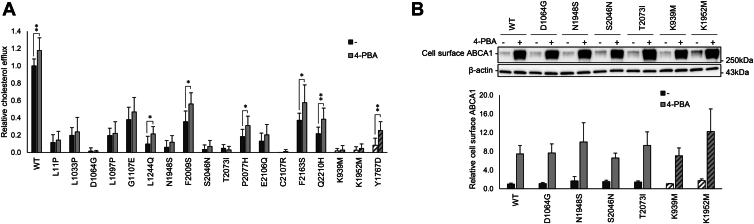
Functional rescue of loss-of-function *ABCA1* variants by 4-PBA treatment. Effect of 4-PBA on functionality of 15 loss-of-function variants and three control variants (striped columns), assessed in transiently transfected HEK293 cells after 20 h incubation with 10 mM 4-PBA or an equal volume of dH_2_O (−). Error bars represent 1 SD. A: Relative cholesterol efflux normalized to mock-treated WT ABCA1 (WT) in five independent experiments. ∗*P* < 0.05, ∗∗*P* < 0.01, ∗∗∗*P* < 0.001, ∗∗∗*P* < 0.0001, one-tailed *t-*test versus mock-treatment. B: Cell surface expression of the ATPase deficient control variants K939M and K1952M, and loss-of-function variants with a suspected ATPase deficiency, normalized to mock-treated WT ABCA1 (WT) in three independent experiments (*P* < 0.05, one-tailed *t-*test vs. mock-treatment). One representative Western blot is shown. ABCA1 was detected using a V5-HRP antibody, and β-actin in cell lysates was used as a loading control.

In our recent functional characterization of variants affecting the extracellular domains of ABCA1, we demonstrated that some of the protein dysfunction of transport-deficient loss-of-function variants could be rescued by the chemical chaperone 4-phenylbutyric acid (4-PBA) (23). Since most of the 15 loss-of-function variants identified herein demonstrated reduced amounts of ABCA1 at the cell surface, there are potential therapeutic benefits by circumventing the dysfunction by treatment with 4-PBA. Consequently, ABCA1 functionality was evaluated after 4-PBA (Sigma-Aldrich) treatment for 20 h in HEK293 cells transiently transfected with 18 loss-of-function variants (15 characterized herein and the three control variants p.K939M, p.K1952M, and p.Y1767D) (Fig. 3A). Although 4-PBA increased the amount of total and cell surface ABCA1 protein for all variants (data not shown), the impact on cholesterol efflux activity was variant dependent, in accordance with our previous work (23). The variants p.L1244Q, p.F2009S, p.P2077H, p.F2163S, and p.Q2210H obtained a significant increase in cholesterol efflux after 4-PBA treatment, similarly to the control variant p.Y1767D, corroborating some residual activity of these variants. The remaining loss-of-function variants did not demonstrate improved functionality with 4-PBA, suggesting that these variants result in a deficient ABCA1 transporter. Moreover, the variants p.D1064G, p.N1948S, p.S2046N and p.T2073I, all located in ATP-binding motifs (signature or Walker A or B), continued an extreme reduction in cholesterol efflux activity despite a marked increase in cell surface ABCA1 after 4-PBA treatment (Fig. 3B). This phenotype is comparable to that of the ATPase deficient controls p.K939M and p.K1952M, suggesting deficient ATPase activity.

### Localization of variants in the ABCA1 protein

**Fig. 4:**
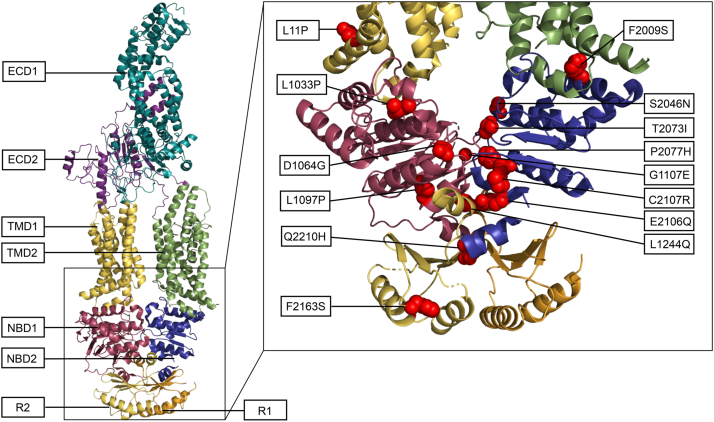
Domain structure of ABCA1 and localization of loss-of-function variants. Each ABCA1 domain structure is distinctly colored for clarity: ECD1 is shown in cyan, ECD2 in purple, TMD1 in yellow, TMD2 in green, NBD1 in red-violet, NBD2 in blue, and the R-domains (R1 and R2) in orange and yellow. The accompanying enhanced image on the right highlights the structural mapping of 14 of the 15 loss-of-function variants specifically located in NBD1 (residues 903–1147), NBD2 (residues 1907–2143), R-domains (residues 1182–1251 and 2155–2220), and the intracellular coupling helices (residues; IH1: 3–20, IH2: 667–673, IH3: 1327–1344 and IH4: 1684–1690). This structural representation is derived from Protein Data Bank entry 7ROQ and was created using PyMOL (The PyMOL Molecular Graphics System, Version 3.1.6.1, Schrödinger, LLC, New York, NY). Because p.N1948 is not depicted in the 7ROQ model, the localization of this residue is shown in supplemental Fig. S4B.

Of the 15 identified loss-of-function variants, one (p.L11P) is localized in one of the intracellular coupling helices (IH1) in TMD1, four (p.L1033P, p.D1064G, p.L1097P and p.G1107E) are in NBD1, seven (p.N1948S, p.F2009S, p.S2046N, p.T2073I, p.P2077H, p.E2106Q and p.C2107R) are in NBD2 and three (p.L1244Q, p.F2163S and p.Q2210H) are in the R domains (Fig. 4 and supplemental Fig. S4A). Reviewing the ABCA1 protein structure revealed that the majority of the NBD loss-of-function variants are located in the interface between NBD1 and NBD2. Specifically, nine (p.L1033P, p.D1064G, p.G1107E, p.N1948S, p.S2046N, p.T2073I, p.P2077H, p.E2106Q and p.C2107R) of the 11 loss-of-function variants in the NBDs are localized in this region, compared to only two functionally normal variants (p.R666Q and p.S1067C) (supplemental Fig. S4B), emphasizing the importance of the structural integrity of this interface region in maintaining ABCA1 functionality.

### Effects of equivalent variants in the two nucleotide-binding domains

**Fig. 5:**
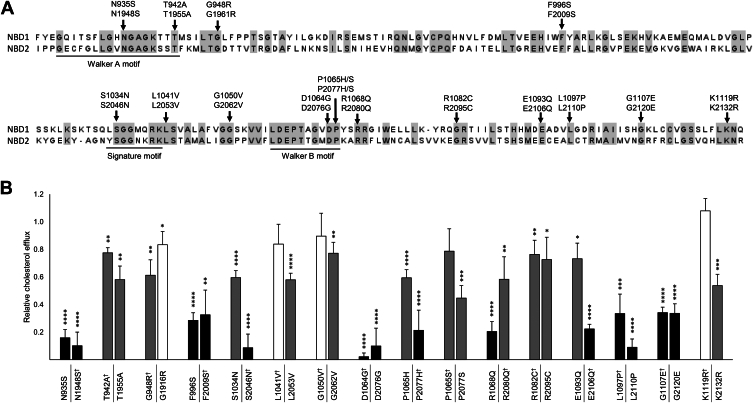
Comparison of the two ABCA1 nucleotide-binding domains. Comparison of sequence and equivalent variants of the two nucleotide-binding domains (NBD1 and NBD2) of ABCA1. A: Sequence alignment of the two NBDs, specifically from residue 923 to 1121 in NBD1, and from residue 1936 to 2134 in NBD2. The alignment was conducted using Jalview with the MUSCLE algorithm (28) and is visualized using a gray color scheme to highlight the conserved residues. Equivalent variants assessed in this study are indicated. B: Relative cholesterol efflux activity of 16 equivalent NBD variant pairs (NBD1|NBD2), normalized to WT ABCA1 in four independent experiments. Variants were assessed to be functionally normal (efflux > 80% of WT, white column), loss-of-function (efflux < 41% of WT, black columns), or of uncertain significance (efflux 41%–80% of WT, gray columns). Error bars represent 1 SD. ∗*P* < 0.05, ∗∗*P* < 0.01, ∗∗∗*P* < 0.001, ∗∗∗∗*P* < 0.0001, *t-*test versus WT ABCA1. ^†^Data already presented in Fig. 1.

Exploring the structural localization of the various variants, we observed that NBD2 had a higher occurrence of loss-of-function variants, with seven out of 14, compared to four out of 21 in NBD1. Since the NBDs display a high degree of sequence homology and share well-conserved motifs, this discrepancy in the incidence of loss-of-function variants raises the question of whether NBD2 is more vulnerable to variation than NBD1. To further explore the impact of missense variants in these domains, we extended our analysis to include 16 additional variants in residues conserved across both NBDs, effectively mirroring the variants of NBD1 and NBD2 in the respective opposite domain (Fig. 5, supplemental Figs. S3 and S5).

Of the 16 equivalent variant pairs, seven showed similar cholesterol efflux in both domains (p.N935S and p.N1948S, p.T942A and p.T1955A, p.F996S and p.F2009S, p.G1050V and p.G2062V, p.D1064G and p.D2076G, p.R1082C and p.R2095C, p.G1107E, and p.G2120E) (Fig. 5B). Five NBD2 equivalents (p.S2046N, p.L2053V, p.P2077H, p.E2106Q, and p.K2132R) were categorized with considerably less efflux than their NBD1 equivalents (p.S1034N, p.L1041V, p.P1065H, p.E1093Q, and p.K1119R, respectively), and the NBD2 variants p.P2077S and p.L2110P were less tolerated than their respective equivalents p.P1065S and p.L1097P, although they ended up in the same functional category. Only two NBD2 equivalents (p.G1916R and p.R2080Q) showed a distinctly increased cholesterol efflux compared to their NBD1 equivalents (p. G948R and p.R1068Q, respectively). Thus, in total, among the 16 equivalent variant pairs, seven had comparable effects on cholesterol efflux in both domains, seven were less tolerated in NBD2, and only two were less tolerated in NBD1, indicating that NBD2 may indeed have a lower tolerance for variation than NBD1.

For further comparison among the NBD equivalent variants, we assessed the cell surface amounts of the equivalent pairs where one or both were characterized as loss-of-function based on cholesterol efflux activities. The results demonstrated similar amounts of cell surface ABCA1 within each equivalent pair (supplemental Fig. S5), indicating a consistent loss-of-function mechanism across the equivalents in each pair.

### Alanine substitution restored functionality of three NBD variants

The three NBD variants p.L1033P, p.T2073I, and p.C2107R were excluded from the NBD equivalent variants experiment as the residues are not conserved across both domains. All three variants exhibited severely reduced functionality, with cholesterol efflux levels below 10% of WT ABCA1. To assess whether these positions would tolerate a different amino acid substitution, we functionally characterized variants in which these amino acids were replaced with alanine (supplemental Figs. S3 and S6). This substitution was much better tolerated at all three positions, recovering more than 50% of the lost cholesterol efflux. The resulting variants p.L1033A, p.T2073A, and p.C2107A demonstrated cholesterol efflux activities of 58 ± 10%, 79 ± 4%, and 89 ± 11%, respectively, indicating that the severe functional deficits were not primarily due to the loss of the native residues at these positions, but rather to the deleterious effects of the substitutions to proline, isoleucine, and arginine.

### Classification of *ABCA1* variants using the ACMG/AMP guidelines

Functional characterization of genetic variants may be applied as evidence under the pathogenic criterion PS3 or the benign criterion BS3 in ACMG/AMP classification if the functional assay is “well-established” (26). The Clinical Genome Resource Sequence Variant Interpretation Working Group recommends that a functional assay may be used as evidence at moderate strength (equivalent to two supporting criteria when used as benign) if the assay reflects the disease mechanism, utilizes sample replicates, and includes normal and null controls in addition to at least 11 variant controls with ACMG/AMP class 1, 2, 4 or 5 (30). We have, in this study, included WT and mock controls, sample replicates as well as 15 variant controls in addition to the well-documented Tangier variant p.W590S. Excluding the results from this study, 58 of the 74 variants assessed were evaluated as class 3 variants (uncertain significance), where more data is needed to determine their clinical consequence (supplemental Table S1). By including the functional characterization in this study into the variant assessment, thus providing evidence for the PS3 or BS3 criteria at moderate strength for 50 variants (15 loss-of-function and 35 functionally normal), the uncertainty of their clinical significance is reduced. This changes the ACMG/AMP class for 12 of the 74 variants evaluated (Table 1).

**Table 1:** *ABCA1* variants for which the ACMG/AMP classification was altered with this study. Functional characterization based on cholesterol efflux (Fig. 1) applied as criteria at moderate strength reclassified the given variants. Additional ACMG criteria applied are also indicated.

Variant	ACMG criteria^a^
Class 3 to 2 when including functionally normal characterization^a^	
p.S1157N	BP4
p.N1185S	-
p.R1341T	BS1
p.R1925Q	BP4
p.R2200Q	BP4
Class 3 to 4 when including loss-of-function characterization^a^	
p.L1033P	PM2, PP3, PP4
p.D1064G	PM1_supporting, PM2, PP3
p.N1948S	PM1_supporting, PM2, PP3
p.T2073I	PM1_supporting, PM2, PP3
p.Q2210H	PM2, PP3, PP4
Class 4 to 5 when including loss-of-function characterization^a^	
p.F2009S	PM2, PM3, PP3, PP4_strong
p.E2106Q	PM2, PM3, PP3, PP4_strong

a Benign criteria weighed as supporting (BP4) or strong (BS1). Pathogenic criteria were weighed as supporting (PP3-4, PM1_supporting), moderate (PM2-3), or strong (PP4_strong). Functionally normal characterization (cholesterol efflux > 80% of WT ABCA1) was applied as BS3_moderate, and loss-of-function characterization (cholesterol efflux < 41% of WT ABCA1) was applied as PS3_moderate. Class 2: likely benign; Class 3: uncertain significance; Class 4: likely pathogenic; Class 5: pathogenic.

## Discussion

In this study, we have functionally characterized 74 *ABCA1* variants, all situated within the protein's intracellular regions. Although the majority of these variants are associated with reduced HDL-C levels, a common feature across many study selection cohorts, our findings showed significant variation in functionality. Fifteen variants displayed cholesterol efflux levels lower than the disease-causing loss-of-function control variant p.W590S, at only 41% of WT ABCA1 activity. In contrast, 35 variants exceeded our functionally normal threshold of 80% of WT ABCA1 efflux, while 24 variants demonstrated cholesterol efflux activity ranging from 41% to 80% of WT ABCA1. These results demonstrate a spectrum of functional impacts among the variants studied.

### Characterization of *ABCA1* loss-of-function variants

We contend that the 15 variants that displayed cholesterol efflux levels below that of the control p.W590S are accurately characterized as loss-of-function, a categorization that holds clinical relevance for patients harboring these genetic variants. Supporting this conclusion, 13 of these 15 variants have been identified in either heterozygous, compound heterozygous, or homozygous forms in patients exhibiting distinct and coherent phenotypes that reinforce the characterization (supplemental Table S3). Notably, the variant p.L1097P (33 ± 14% of WT ABCA1 cholesterol efflux) was in 2015 reported homozygous in a 17-year-old male patient diagnosed with Tangier disease (31). This patient exhibited lipid accumulation in the tonsils, splenomegaly, and severely reduced HDL-C (0.05 mmol/L) with undetectable ApoA1 in plasma. Further, the variants p.S2046N (9 ± 10%), p.P2077H (21 ± 15%) (32), p.F2009S (33 ± 18%), and p.D1099Y (46 ± 15%) (33) and p.W590X (34) and the novel p.E2106Q (22 ± 3%), were identified as compound heterozygous in individuals with severely reduced plasma HDL-C and ApoA1 levels. This clinical evidence solidifies the validity of our loss-of-function categorization.

### Characterization of functionally normal *ABCA1* variants

Based on our findings, we are also confident that variants displaying cholesterol efflux levels at or above 80% of WT ABCA1 can be appropriately characterized as functionally normal, with negligible clinical relevance for individuals carrying these genetic variants. We established this threshold based on the efflux activities of the two variants p.I883M (84 ± 8%) and p.V2244I (75 ± 11%), both involving a substitution between two hydrophobic amino acids, in addition to previous findings (23). The variant p.I883M is present at a high allele frequency, and p.V2244I is found in multiple other vertebrate species, suggesting no significant impact on ABCA1 functionality. In addition to p.I883M, five other variants evaluated in this study exhibit high allele frequencies in the population and were classified as likely benign or benign under ACMG/AMP guidelines. These include p.E868K (105 ± 9%), p.C887F (87 ± 9%), p.L1041V (84 ± 14%), p.E1172D (74 ± 9%), and p.S1255R (85 ± 4%). These variants, except for p.E1172D, exceeded the 80% cholesterol efflux threshold. While there may be uncertainty regarding the classification of p.V2244I as likely benign or benign due to different tolerances of amino acids across vertebrate species, the allele frequency of p.E1172D of 17%, which is uncommon among pathogenic variants, suggests that individuals with variants demonstrating cholesterol efflux levels as low as 74% of WT ABCA1 might exhibit a fairly normal phenotype without significantly affected HDL-C levels.

### Characterization of *ABCA1* variants of uncertain significance

The characterization of loss-of-function or normal variants based on functional analyses is relatively uncomplicated when their activities are either close to WT or below a negative control. However, the characterization of variants with activities between these two thresholds is less certain, herein variants presenting cholesterol efflux between 41% and 80% of WT ABCA1. The cutoff of 41% corresponds to the efflux activity of the control variant p.W590S, which was selected based on previous experiments where the variant showed the highest cholesterol efflux level among several loss-of-function controls (23). However, there is no evidence that variants that marginally outperform this negative control lack clinical significance. In fact, five of the variants (p.G851R, p.D1099Y, p.R1680W, p.R1901S and p.R2080Q) placed in the variants of uncertain significance category based solely on the cholesterol efflux, were classified as likely pathogenic according to the ACMG/AMP guidelines. These are all in well-conserved residues and present at extremely low frequencies in the normal population.

The variants p.G851R (46 ± 9%) and p.R1680W (56 ± 14%) have both been reported homozygous, and p.D1099Y (46 ± 15%) and p.R2080Q (58 ± 16%) were found compound heterozygous with p.F2009S (33 ± 18%) and c.-93+2dup, respectively, in patients with Tangier disease (33, 35, 36, 37). The variant p.R1901S had the highest cholesterol efflux among the variants classified as likely pathogenic by ACMG/AMP, with an efflux of 66 ± 13% of WT ABCA1. This variant was reported compound heterozygous with the *ABCA1* frameshift variant c.479del (p.H160Pfs∗14) in an individual with a plasma HDL-C level of 0.18 mmol/l (38). The patient had a tonsillectomy at a young age and no reported symptoms of Tangier disease, and although the HDL-C was severely reduced, the level was generally higher than seen in most Tangier patients (6). Considering the recessive nature of Tangier disease, which necessitates loss-of-function variants in both *ABCA1* alleles for clinical manifestation, and the fact that heterozygous carriers of loss-of-function variants generally exhibit only a 40%–50% reduction in HDL levels (8), the severe reduction in HDL-C levels observed in the p.R1901S patient would likely require functionally abnormal *ABCA1* variants in both alleles, or other genetic factors to explain the phenotype. This indicates that while the p.R1901S variant is indeed likely pathogenic, it may not be as detrimental as other well-established variants associated with Tangier disease.

However, the clinical evidence from patients with Tangier disease, caused by variants such as p.G851R, p.D1099Y, p.R1680W, and p.R2080Q, suggests that a higher disease-causing threshold may apply to more accurately identify variants that are true loss-of-function. In this regard, using the highest cholesterol efflux level among these four variants, a 58% efflux relative to WT ABCA1, as observed for p.R2080Q, may represent a more accurate cutoff. This approach is recommended when applying the functional characterization criterion in ACMG/AMP classification (30). This new threshold would not only reaffirm the classification of these four variants but also characterize three additional variants (p. G1346E, p.Y2178H, and p.Y2206D) as loss-of-function. Consequently, these seven variants, currently characterized as variants of uncertain significance in our experiments and displaying cholesterol efflux levels at or below 58%, are likely loss-of-function and will most likely exhibit clinical symptoms and impact HDL-C levels in patients with these genetic variants.

### Reclassification of ACMG class 3 variants using our functional characterization as evidence

Based on the ACMG/AMP guidelines, excluding additional data from this study, 58 of the 74 variants evaluated in this study were classified as class 3 variants with uncertain significance (supplemental Table S1), highlighting the need for additional data to determine their pathogenicity. Class 3 variants are abundant in clinical variant assessment and do not qualify to initiate hereditary cascade screening. These variants are thus regularly not communicated to the referring physician or patient. When including the functional characterization from this study as evidence in the ACMG/AMP classification, 10 of these class 3 variants are reclassified (Table 1). Additionally, our findings will provide the necessary evidence for reclassifying six more variants (p.L11P, p.G1107E, p.L1244Q, p.F2163S, p.Y2178H, and p.Y2206D) as likely pathogenic, if detected in a patient displaying low HDL levels in a future diagnostic setting.

### Mechanism for loss-of-function variants affecting the intracellular domains of ABCA1

Reduced cell surface localization is a recognized loss-of-function mechanism for disease-causing *ABCA1* variants (3). Eight of the 15 variants identified as loss-of-function in this study exhibited less than 50% of WT ABCA1 cell surface protein levels, which at least partially explains their impact on cholesterol efflux. Notably, the variant p.L11P is located in a sequence reported to act as a Golgi exit signal (39), which may be the reason for its significantly reduced cell surface protein levels. Furthermore, the presence of a proline residue in an alpha helix, known to disrupt this secondary structure, could also be a contributing factor to the observed reduction in cell surface levels. This variant was one of only two that presented less than 50% cell surface ABCA1 compared to WT ABCA1 when adjusted for total ABCA1 levels, indicating that most of the intracellular variants demonstrating cell surface deficiency are primarily exposed to an increased degradation rate rather than transport inefficiency. In contrast, in our recent work on functionally characterizing variants affecting the extracellular domains of ABCA1, all of the identified loss-of-function variants (n = 12) presented as transport deficient (23). Although the number of variants is low, this suggests that the subcellular transport of ABCA1 is more likely to be affected by variants in the extracellular rather than the intracellular domains of the protein.

Another mechanism for loss-of-function of *ABCA1* variants, especially relevant for the intracellular domains, is loss of ATPase activity. Loss of intracellular ATPase function completely inhibits the cholesterol efflux activity of ABCA1 without inducing protein degradation, as demonstrated by the ATPase hindering controls p.K939M and p.K1952M (20). Whereas seven of the loss-of-function variants in this study showed extremely low cholesterol efflux of 10% or less compared to WT ABCA1, only p.N1948S retained cell surface protein levels comparable to WT ABCA1 and p.K939M. The variant p.N1948S is indeed located in the Walker A motif of NBD2, indicating that the variant results in deficient ATPase function. Despite a somewhat reduced surface amount for the variants p.D1064G, p.S2046N and p.T2073I, which also resulted in extremely reduced cholesterol efflux, their localization in the Walker B or signature motifs suggests that they also hamper the ATPase activity. Accordingly, the cholesterol efflux activities of these four variants were unaffected following treatment with the chemical chaperone 4-PBA, although their levels of cell surface ABCA1 were increased, as for the ATPase deficient controls p.K939M and p.K1952M.

### Localization of intracellular loss-of-function variants

Out of the 15 loss-of-function variants, 11 are situated in one of the two NBDs, three are found in the R domains, and one variant, L11P, is localized in an intracellular hinge region of TMD1. Of the 11 variants in the NBDs, 10 are in residues 100% conserved across the 28 species in the multiple sequence alignment (supplemental Fig. S2). However, several functionally normal variants are also in residues with a high degree of conservation, underlining that conservation alone is not sufficient to determine pathogenicity. Further indications could be the severity of amino acid substitutions, where the majority of the loss-of-function variants have larger changes in amino acid properties than many of the changes among the normal variants. Furthermore, a major difference between the loss-of-function variants and the normal variants is evident in their localizations in the tertiary structure. Out of the 11 loss-of-function variants found in the NBDs, nine are localized in the interface region between the two domains, while only two of the functionally normal variants are found in this region. It should be noted that only 23 of the 35 variants characterized as functionally normal were possible to include in the 7ROQ ABCA1 protein model, rendering 12 functionally normal variants not mapped in the tertiary structure (9). However, the majority of these 12 variants are also absent in other protein models (19, 40), indicating that the region in which these amino acids reside may be highly flexible or intrinsically disordered, and thus likely not critical for the interaction between the two NBDs. This finding concurs with earlier publications, which have demonstrated that amino acids residing in the interfacing regions are more highly conserved than those in surrounding areas (41), and that the ABCA1 ATP-binding sites are located at the NBD-NBD interface (18), signifying that the region between the two domains is very important for ABCA1 functionality.

Of the 14 R domain-located variants assessed in this study, three (p.L1244Q, p.F2163S, and p.Q2210H) were characterized as loss-of-function variants. The R domains of ABCA1 lack thorough research; however, one recent study proposed that these domains cross over to form a domain-swapped latch, clamping the two NBDs together (40). It is reasonable to believe that this structure is important for the conformational arrangements of ABCA1, supported by the fact that this domain swapping seems to be conserved in all transporters of the ABCA subfamily (40). Thus, it is plausible that the three loss-of-function variants located in these domains interfere with the latch, particularly the variants p.L1244Q and p.Q2210H, which are located in the conserved crossover/crossback sequences (residues 1229–1254 and 2198–2223).

### Variant tolerability of the two NBDs of ABCA1

The majority of the loss-of-function variants identified in this study affect one of the two NBDs, highlighting not only the size of these domains, but also their critical functions. The amino acid sequences of the two NBDs are highly conserved among ABC transporters (42) and among ABCA1 of different species. The sequence is also quite conserved between the two domains with 39% sequence similarity, implying that they both have equal roles in ABCA1 ATPase activity. Herein we characterized four out of 21 NBD1 variants and seven out of 14 NBD2 variants as loss-of-function. This disparity raised the question of whether NBD2 has a lower tolerance for variation compared to NBD1. We therefore expanded the functional analysis to include all equivalent variants in the respective opposite NBD of the variants studied herein, where the amino acid residue is conserved in both domains. Of the 16 equivalent variant pairs, seven demonstrated a similar effect on cholesterol efflux in both domains, seven were less tolerated in NBD2 and only two were less tolerated in NBD1. Thus, in many cases we can expect the same effect on ABCA1 function of equivalent variants in both NBDs, although NBD2 seems to be somewhat less tolerable to amino acid substitutions.

A recent study has shown that ABCA1 contains two symmetrical ATP-binding sites composed of the Walker A and B motifs from one NBD and the signature motif from the other NBD (18). This indicates that both NBDs play an equal part in ATP-binding, which has also been demonstrated by others (17, 43), contrary to our finding that NBD2 shows a lower tolerance to variation than NBD1. However, the latter studies also suggested differences between the two NBDs in ATPase related activities, where one showed that NBD1 has a higher ATPase activity than NBD2 (43), and the other observed occlusion of ADP after ATP-hydrolysis to a higher degree in NBD2 than in NBD1 (17). Although these studies were performed on purified soluble single NBDs, which could act differently than the NBDs in the native ABCA1 protein, they might indicate that the difference in variance tolerability between the two domains is due to differences in ATPase activity. Of the variants excluded from our study due to previous functional characterization, seven of eight NBD1 variants and two of four NBD2 variants lost substantial functionality (29, 44, 45, 46, 47, 48, 49), which together with our results validates the low variance tolerability of the NBDs, with NBD2 possibly being more vulnerable.

The Walker A and B motifs and the signature motif are evident as conserved sequences in the sequence alignment of the two NBDs. Several variants located in the Walker A and B motifs have been functionally characterized in previous studies, indicating that variation in these sequences is generally not well tolerated (21, 44, 45, 46, 47, 48, 50). We evaluated nine variants located in these three motifs (excluding equivalents), six of which were assessed to be loss-of-function (p.N1948S in the Walker A motif; p.D1064G, p.T2073I and p.P2077H in the Walker B motif; p.L1033P and p.S2046N in the signature motif). The remaining three variants, located in NBD1, were characterized as functionally normal or of uncertain significance. The signature motif variant p.M1037T was functionally normal, and although both the Walker A variant p.T942A (77 ± 4%) and the Walker B variant p.P1065S (79 ± 16%) were characterized as uncertain in this study, their efflux activities were very close to the 80% cutoff for functionally normal variants. These three variants change the residue from polar to non-polar or vice versa and still retain almost full functionality. Thus, our results support the notion that most of the variants in these motifs deteriorate ABCA1 function, although they do tolerate some variation.

## Conclusion

The functional characterization in this study demonstrates a spectrum of functional impact among 74 *ABCA1* variants previously reported and associated with HDL deficiency. In accordance with ACMG/AMP guidelines, the result has reclassified five *ABCA1* variants from class 3 (uncertain significance) to class 2 (likely benign), confirmed the pathogenic status of eight variants, and shifted five more from class 3 to class 4 (likely pathogenic). In addition, our findings empower geneticists to confidently reclassify six more variants currently in class 3 to class 4, should they encounter patients exhibiting strong clinical phenotypes associated with these genetic variants. This highlights the indispensability of functional characterization in aiding clinical variant assessment, improving the diagnostic yield. Further, functional studies of naturally occurring variants contribute to improving the understanding of how these proteins function, paving the way for future therapeutic targets.

## Data availability

All data supporting the findings of this study are available within this article and its supplementary information.

## Supplemental data

This article contains supplemental data (24, 26, 28, 31, 32, 33, 35, 36, 37, 38, 51, 52, 53, 54, 55, 56, 57, 58, 59, 60, 61, 62, 63, 64, 65, 66, 67, 68, 69, 70, 71, 72, 73, 74, 75, 76, 77, 78).

## Conflict of interest

The authors declare that they have no conflicts of interest with the contents of this article.
