# Supplementary material for: Functional characterization of genetic variants affecting the intracellular domains of ATP-binding cassette transporter A1 (ABCA1)
Source: J Lipid Res. 2025 Jul 3;66(8):100854. doi: 10.1016/j.jlr.2025.100854 (PMC12341595; doi:10.1016/j.jlr.2025.100854)
Supplement: Supplemental Data S1 [file mmc1.pdf]

Supplemental Data S1

Functional characterization of genetic variants affecting the intracellular domains of ATP-binding cassette transporter A1 (ABCA1)

Marianne Teigen, Åsa Schawlann Ølnes, Katrine Bjune, Martin Prøven Bogsrud and Thea Bismo Strøm

Supplemental Table S1: Missense *ABCA1* variants functionally characterized in this study. *ABCA1* variants are annotated at protein and nucleotide level. An *in silico* prediction of pathogenicity is given by a REVEL score (51). Allele frequencies of the variants were obtained from the Genome Aggregation Database (https://gnomad.broadinstitute.org/) (gnomAD) v.4.1.0. Pathogenicity class of the variants evaluated according to the ACMG/AMP guidelines are shown along with the criteria applied (26).

Variant				ACMG/AMP		
Protein level	Nucleotide level	REVEL score[a]	gnomAD[b]	Class[c]	Criteria[d]	Ref.
p.L11P	c.32T>C	0.898	1/584009 (NFE)	3	PM2, PP3	(36)
p.R666Q	c.1997G>A	0.575	1/6861 (NFE)	3	PM2	(52)
p.R672Q	c.2015G>A	0.892	1/6084 (MID)	3	PM2, PP3	(53)
p.D677E	c.2031C>A	0.498	1/1614170 (All)	3	PM2	(54)
p.P847L	c.2540C>T	0.817	1/294997 (NFE)	3	PM2, PP3	(55)
p.G851R	c.2551G>A	0.896	1/30353 (SA)	4	PM2, PM3_sup, PP3, PP4_str	(56)
p.E868K	c.2602G>A	0.367	1/62 (AFR)	1	BA1	(57)
p.I883M	c.2649A>G	0.156	1/2 (EA)	1	BA1, BP4	(58)
p.C887F	c.2660G>T	0.488	1/143 (EA)	1	BA1	(54)
p.T942A	c.2824A>G	0.857	-	3	PM1_sup, PM2, PP3	(59)
p.G948R	c.2842G>A	0.935	1/131114 (NFE)	3	PM2, PP3	(32)
p.R965C	c.2893C>T	0.807	1/1508 (MID)	3	PP3	(60)
p.M968T	c.2903T>C	0.694	1/5198 (NFE)	3	PM2	(61)
p.R972W	c.2914C>T	0.904	1/22693 (NFE)	3	PM2, PP3	(52)
p.V983M	c.2947G>A	0.879	1/74936 (AFR)	3	PM2, PP3	(62)
p.A1010V	c.3029C>T	0.556	1/6084 (MID)	3	PM2	(54)
p.D1018G	c.3053A>G	0.841	1/124 (AJ)	3	PP3	(52)
p.L1033P	c.3098T>C	0.974	1/30011 (AMR)	3	PM2, PP3, PP4	(36)
p.M1037T	c.3110T>C	0.915	-	3	PM2, PP3	(63)
p.L1041V	c.3121C>G	0.688	1/178 (EA)	1	BA1	(55)

p.G1050V	c.3149G>T	0.817	1/1180032 (NFE)	3	PM2, PP3	(24)
p.D1064G	c.3191A>G	0.960	1/393340 (NFE)	3	PM1_sup, PM2, PP3	(56)
p.P1065S	c.3193C>T	0.880	1/1614188 (All)	3	PM1_sup, PM2, PP3	(64)
p.S1067C	c.3200C>G	0.829	1/60026 (AMR)	3	PM2, PP3	(24)
p.R1079Q	c.3236G>A	0.916	1/393343 (NFE)	3	PM2, PP3	Novel
p.R1082C	c.3244C>T	0.916	1/5335 (SA)	3	PM2, PP3	(65)
p.L1097P	c.3290T>C	0.913	-	4	PM2, PP3, PP4_str	(31)
p.D1099Y	c.3295G>T	0.887	1/90774 (NFE)	4	PM2, PM3, PP3, PP4_str	(33)
p.G1107E	c.3320G>A	0.936	-	3	PM2, PP3	(52)
p.K1119R	c.3356A>G	0.719	-	3	PM2, PP3	(52)
p.S1157N	c.3470G>A	0.347	1/1496 (EA)	3	BP4	(52)
p.E1172D	c.3516G>C	0.332	1/6 (AFR)	1	BA1, BP4	(66)
p.S1181F	c.3542C>T	0.605	1/263 (EF)	3	-	(60)
p.A1182T	c.3544G>A	0.167	1/937 (NFE)	3	BP4	(56)
p.N1185S	c.3554A>G	0.707	1/2580 (AFR)	3	-	(53)
p.R1195Q	c.3584G>A	0.401	1/1319 (EA)	3	-	(52)
p.G1216V	c.3647G>T	0.904	1/21344 (EF)	3	PM2, PP3	(64)
p.L1244Q	c.3731T>A	0.969	-	3	PM2, PP3	(67)
p.S1255R	c.3763A>C	0.320	1/306 (AFR)	2	BS1, BP4	(52)
p.Q1279K	c.3835C>A	0.581	-	3	PM2	(57)
p.G1321A	c.3962G>C	0.811	1/60000 (AMR)	3	PM2, PP3	(63)
p.R1341T	c.4022G>C	0.529	1/354 (AFR)	3	BS1	(60)
p.R1344Q	c.4031G>A	0.769	1/45544 (SA)	3	PM2, PP3	(68)
p.G1346E	c.4037G>A	0.833	1/423 (AJ)	3	PP3	(36)
p.R1680Q	c.5039G>A	0.871	1/1599 (NFE)	3	PM5_sup, PP3	(60)
p.R1680W	c.5038C>T	0.881	1/12806 (EF)	4	PM2, PP3, PP4_str	(37)
p.R1879T	c.5636G>C	0.704	-	3	PM2, PP3	(53)
p.E1891A	c.5672A>C	0.610	1/6060 (MID)	3	PM2	(56)
p.R1897W	c.5689C>T	0.495	1/18709 (AFR)	3	PM2	(69)
p.R1901S	c.5703A>C	0.668	-	4	PM2, PM3, PP4_mod	(38)
p.I1911M	c.5733C>G	0.817	1/2909 (EF)	3	PP3	(53)

p.I1920L	c.5758A>T	0.296	-	3	PM2, BP4	(52)
p.R1925Q	c.5774G>A	0.436	1/94 (EF)	3	BP4	(70)
p.N1948S	c.5843A>G	0.928	-	3	PM1_sup, PM2, PP3	(71)
p.H1985Y	c.5953C>T	0.765	1/9148 (NFE)	3	PM2, PP3	(52)
p.F2009S	c.6026T>C	0.933	-	4	PM2, PM3, PP3, PP4_str	(33)
p.R2030W	c.6088C>T	0.818	1/45535 (SA)	3	PM2, PP3	(52)
p.S2046N	c.6137G>A	0.776	-	5	PM2, PM3, PM5_sup, PP3, PP4_str	(32)
p.T2073I	c.6218C>T	0.835	-	3	PM1_sup, PM2, PP3	(36)
p.P2077H	c.6230C>A	0.880	-	5	PM1_sup, PM2, PM3, PP3, PP4_str	(32)
p.R2080Q	c.6239G>A	0.870	1/393327 (NFE)	4	PM2, PM3, PP3, PP4_str	(35)
p.E2106Q	c.6316G>C	0.955	-	4	PM2, PM3, PP3, PP4_str	Novel
p.C2107R	c.6319T>C	0.994	1/3557 (EF)	3	PP3	(53)
p.V2143I	c.6427G>A	0.402	1/196668 (NFE)	3	PM2	(52)
p.F2163S	c.6488T>C	0.832	-	3	PM2, PP3	(72)
p.P2164S	c.6490C>T	0.612	1/5693 (SA)	3	PM2	(52)
p.R2173W	c.6517C>T	0.581	1/18214 (SA)	3	PM2	(52)
p.N2174T	c.6521A>C	0.168	1/59000 (NFE)	3	PM2, BP4	(52)
p.Y2178H	c.6532T>C	0.929	-	3	PM2, PP3	(57)
p.Q2196H	c.6588G>C	0.428	1/2089 (EF)	3	-	(38)
p.R2200Q	c.6599G>A	0.197	1/1184 (AJ)	3	BP4	(52)
p.Y2206D	c.6616T>G	0.885	-	3	PM2, PP3	(73)
p.Q2210H	c.6630G>C	0.781	-	3	PM2, PP3, PP4	(74)
p.V2244I	c.6730G>A	0.189	1/433 (MID)	3	BP4	(72)

[a] REVEL scores from 0 to 1, with a higher score indicating a greater likelihood of the variant being deleterious (51). [b] The highest allele frequency among the populations: African (AFR), Amish, Admixed American (AMR), Ashkenazi Jewish (AJ), East Asian (EA), South Asian (SA), Non-Finnish European (NFE), European Finnish (EF), Middle Eastern (MID) or all of the above (All). [c] Classification of pathogenicity according to the guidelines from The American College of Medical Genetics and Genomics and The Association for Molecular Pathology (ACMG/AMP). Class 1: benign; Class 2: likely benign; Class 3: uncertain significance; Class 4: likely pathogenic; Class 5: pathogenic. [d] Pathogenic criteria weighed as supporting (PP3-4, PM1-5_sup), moderate (PM2-3, PP4_mod) or strong (PP4_str); benign criteria weighed as supporting (BP4), strong (BS1) or stand-alone (BA1). Ref.: Reference. Novel: identified at Unit for Cardiac and Cardiovascular Genetics, Oslo University Hospital.

Supplemental Table S2: Mutagenesis primers

The primers used to generate *ABCA1* variants. Only the forward primers with mutated bases underlined are listed unless otherwise specified.

Primer	Sequence 5’-3’
K1952M	GGGGCTGGAATGTCATCAACTTTC
L11P	GGTTGCTGCCGTGGAAGAACC
R666Q	GAGAAGGAGGCACAGCTGAAAGAGACC
R672Q	GAGACCATGCAGATCATGGGC
D677E	CATGGGCCTGGAAAACAGCATAC
P847L	GCTGTCTTTCTAGGCCAGTACGG
G851R	GGCCAGTACAGAATTCCCAGGCCC
E868K	CCTACTGGTTTGGCAAGGAAAGTGATGAG
I883M	CCAGAAGAGAATGTCAGAAATCTGCATGGAGGAGG
C887F	TCAGAAATCTTCATGGAGGAGGAACCCACCC
T942A	GGGAAGACGACCGCCATGTCAATCC
G948R	ATCCTGACCAGGTTGTTCCCCCCG
R965C	CCTGGGAAAAGACATTTGCTCTGAGATGAGCACC
M968T	CGCTCTGAGACGAGCACCATC
R972W	GAGCACCATCTGGCAGAACCTG
V983M	CCCCAGCATAACATGCTGTTTGACATGCTG
A1010V	CACGTGAAGGTGGAGATGGAG
D1018G	CAGATGGCCCTGGGTGTTGGTTTG
L1033P	ACAAGCCAGCCGTCAGGTGGA
M1037T	GTCAGGTGGAACGCAGAGAAAG
L1041V	CAGAGAAAGGTATCTGTGGCC
G1050V	CCTTTGTCGGGGTATCTAAGGTTG
D1064G	GCTGGTGTGGGCCCTTACTCC
P1065S	GGTGTGGACTCTTACTCCCGC
S1067C	GGACCCTTACTGCCGCAGGGGAATATG
R1079Q	CTGAAATACCAACAAGGCCGC
R1082C	CGACAAGGCTGCACCATTATTC
L1097P	GAAGCGGACGTCCCGGGGGACAGGATTG
D1099Y	GTCCTGGGGTACAGGATTGCC
G1107E	CATCTCCCATGAGAAGCTGTGC
K1119R fwd.[a]	CCTGTTTCTGCGCAACCAGCTGGGAACAGGC
K1119R rev.[a]	GAGGAGCCCACACAGCACAGC
S1157N	CCTGAAAAAGGAGGACAATGTTTCTCAGAGCAGTTCTGATGC
E1172D	AGCGACCATGACAGTGACACG
S1181F	CGCTGACCATCGATGTCTTTGCTATCTCC
A1182T	CGCTGACCATCGATGTCTCTACTATCTCCAACC
N1185S	GTCTCTGCTATCTCCAGCCTCATCAGGAAGC
R1195Q	GTGTCTGAAGCCCAGCTGGTGGAAGAC
G1216V	GCTAAGGAGGTAGCCTTTGTG
L1244Q	GAGACGACCCAGGAAGAAATATTCCTCAAGGTGG
S1255R	CAAGGTGGCCGAAGAGCGTGGGGTGGATG
Q1279K	GGGGACAAGAAGAGCTGTCTT
G1321A	CCAGGTGAAAGCCTGGAAACTTAC
R1341T	GCTAATTGCCACACGGAGTCGG
R1344Q	GCCAGACGGAGTCAGAAAGGATTTTTTGCTC
G1346E	GCCAGACGGAGTCGGAAAGAATTTTTTGCTCAG
R1680Q	CTGATCCAGGAGCAGGTCAGCAAAGC
R1680W	ATCCAGGAGTGGGTCAGCAAA
R1879T	CATCAGGCCCACACCTGTAAATGC
E1891A	CCTCTGAATGATGCAGATGAAGATGTGAGGCGGG

R1897W	GATGTGAGGTGGGAAAGACAG
R1901S	GGCGGGAAAGACAGAGCATTCTTGATGG
I1911M	GGTGGAGGCCAGAATGACATGTTAGAAATC
I1920L	GGAGTTGACGAAGTTATATAGAAGGAAGCGGAAGCCTGC
R1925Q	AGAAGGAAGCAGAAGCCTGCT
N1948S	CTGGGAGTTAGTGGGGCTGGA
H1985Y	CCATGAAGTATATCAGAACATGGGCTACTGCCC
F2009S	CACGTGGAGTCCTTTGCCCTT
R2030W	GTGGGCGATTTGGAAACTGGGC
S2046N	GCTGGTAACTATAATGGAGGCAACAAACGC
T2073I	GAACCCACCATAGGCATGGATCCC
P2077H	GGCATGGATCACAAAGCCCGG
R2080Q	CCCAAAGCCCAGCGGTTCTTG
E2106Q	GTATGGAACAATGTGAAGCTCTTTGCACTAGGATGGC
C2107R	ATGGAAGAACGTGAAGCTCTTTGC
V2143I fwd. [a]	TACAATAGTTATTCGAATAGCAGGGTC
V2143I rev. [a]	TAACCATCTCCAAACCTATTTTTAG
F2163S	GGACTTGCATCTCCTGGAAGTG
P2164S	GGACTTGCATTTTCTGGAAGTGTTC
R2173W	GAGAAACACTGGAACATGCTAC
N2174T	GAAACACCGGACCATGCTACAATACC
Y2178H	ATGCTACAACACCAGCTTCCA
Q2196H	GCATCCTCTCCCACAGCAAAAAGCG
R2200Q	CCCAGAGCAAAAAGCAACTCCACATAG
Y2206D	CCACATAGAAGACGACTCTGTTTCTC
Q2210H	CTCTGTTTCTCACACAACACTTGACC
V2244I	GTAGTGGACATTGCAGTTCTC
N935S	CTTCCTGGGCCACAGTGGAGCGGGGAAG
F996S	GAACACATCTGGTCCTATGCCCGCTTG
S1034N	CAAGCCAGCTGAATGGTGGAATGC
P1065H	GGTGTGGACCATTACTCCCGC
R1068Q fwd. [a]	CCCTTACTCCCAGAGGGGAATATGGGAG
R1068Q rev. [a]	TCCACACCAGCTGTGGGTTCA
E1093Q fwd.	CATGGATCAAGCGGACGTCCTGGGGGACAGGATTGC
E1093Q rev.	CGTCCGCTTGATCCATGTGGTGTGTAGAGAGAATAATGGTGC
T1955A	GGGGCTGGAAAATCATCAGCTTTCAAGATG
G1961R	GATGTTAACACGAGATACCACTGTTACCAGAGGAG
L2053V	CAACAAACGCAAGGTCTCTACAGCCATGG
G2062V fwd.	ATCGGCGTGCCTCCTGTGGTGTTTCTGGATGAACC
G2062V rev.	CAGGAGGCACGCCGATCAAAGCCATGGCTGTAGAGAG
D2076G	CACAGGCATGGGTCCCAAAGCC
P2077S	CAGGCATGGATTCCAAAGCCCGG
R2095C	GTCAAGGAGGGGTGTTCAGTAGTGC
L2110P fwd.	GTGAAGCTCCTTGCACTAGGATGGCAATCATGGTCAATGG
L2110P rev.	GCCATCCTAGTGCAAGGAGCTTCACATTCTTCCATACTATGAGATG
G2120E	CATGGTCAATGAAAGGTTCAGGTGCC
K2132R	GCCTTGGCAGTGTCCAGCATCTAAGAAATAGGTTTGG
L1033A	CAAAACAAGCCAGGCGTCAGGTGGAATG
T2073A	GATGAACCCACCGCAGGCATGGATC
C2107A	GTATGGAAGAAGCTGAAGCTCTTTGCACTAGG

[a] Used with the Q5® Site-Directed Mutagenesis Kit (New England Biolabs). Fwd.: forward. Rev.: reverse.

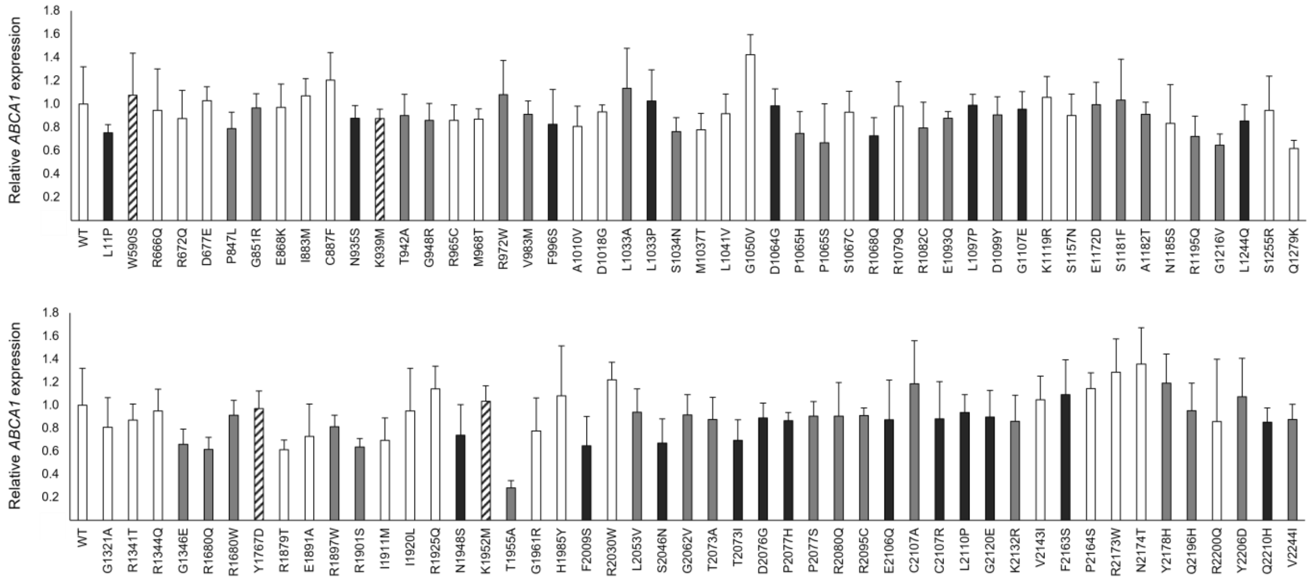

Supplemental Figure S1: mRNA expression of overexpressed *ABCA1*. RNA was isolated from HEK293 cells transiently transfected with *ABCA1* negative control variants (striped columns) and variants characterized as functionally normal (white columns), loss-of-function (black columns) or of uncertain significance (gray columns). Isolated RNA was transcribed to cDNA, which was analyzed using PrimeTime Predesigned qPCR Assay primers (Integrated DNA Technologies, Coralville, IA) for ABCA1 (Hs.PT.58.27452429) and glyceraldehyde-3-phosphate dehydrogenase (GAPDH) (Hs.PT.39a.22214836). *ABCA1* mRNA amounts were determined and normalized to the housekeeping gene *GAPDH* by the $2^{-\Delta\Delta Ct}$ method (75) and again normalized to WT ABCA1 (WT) in three independent experiments. Error bars represent 1 SD.

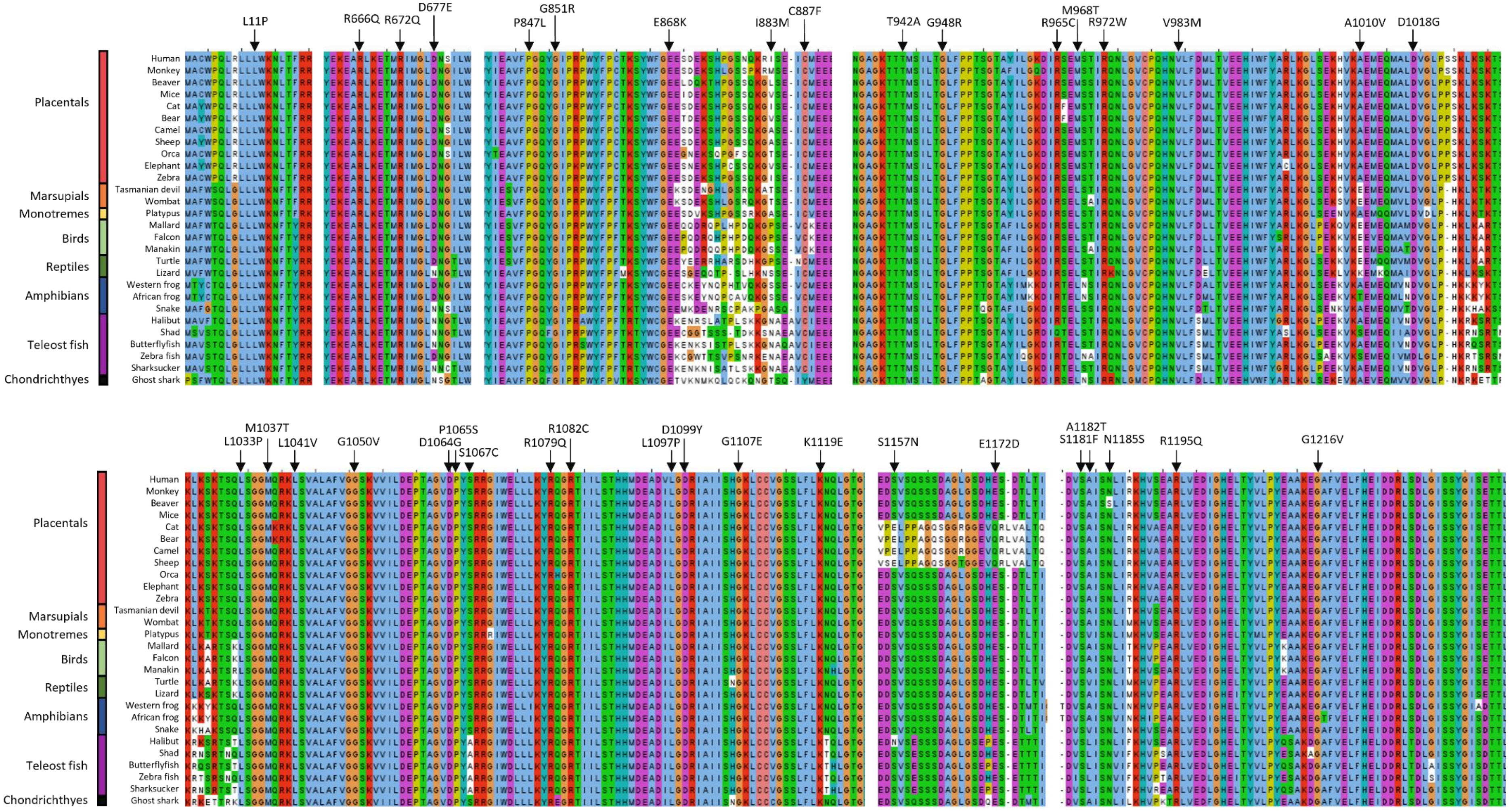
L11P
R666Q
R672Q
D677E
P847L
G851R
E868K
I883M
C887F
T942A
G948R
R965C
M968T
R972W
V983M
A1010V
D1018G
Placentals
Marsupials
Monotremes
Birds
Reptiles
Amphibians
Teleost fish
Chondrichthyes
Human
Monkey
Beaver
Mice
Cat
Bear
Camel
Sheep
Orca
Elephant
Zebra
Tasmanian devil
Wombat
Platypus
Mallard
Falcon
Manakin
Turtle
Lizard
Western frog
African frog
Snake
Halibut
Shad
Butterflyfish
Zebra fish
Sharksucker
Ghost shark
L1033P
M1037T
L1041V
G1050V
D1064G
P1065S
S1067C
R1079Q
R1082C
L1097P
D1099Y
G1107E
K1119E
S1157N
E1172D
S1181F
A1182T
N1185S
R1195Q
G1216V

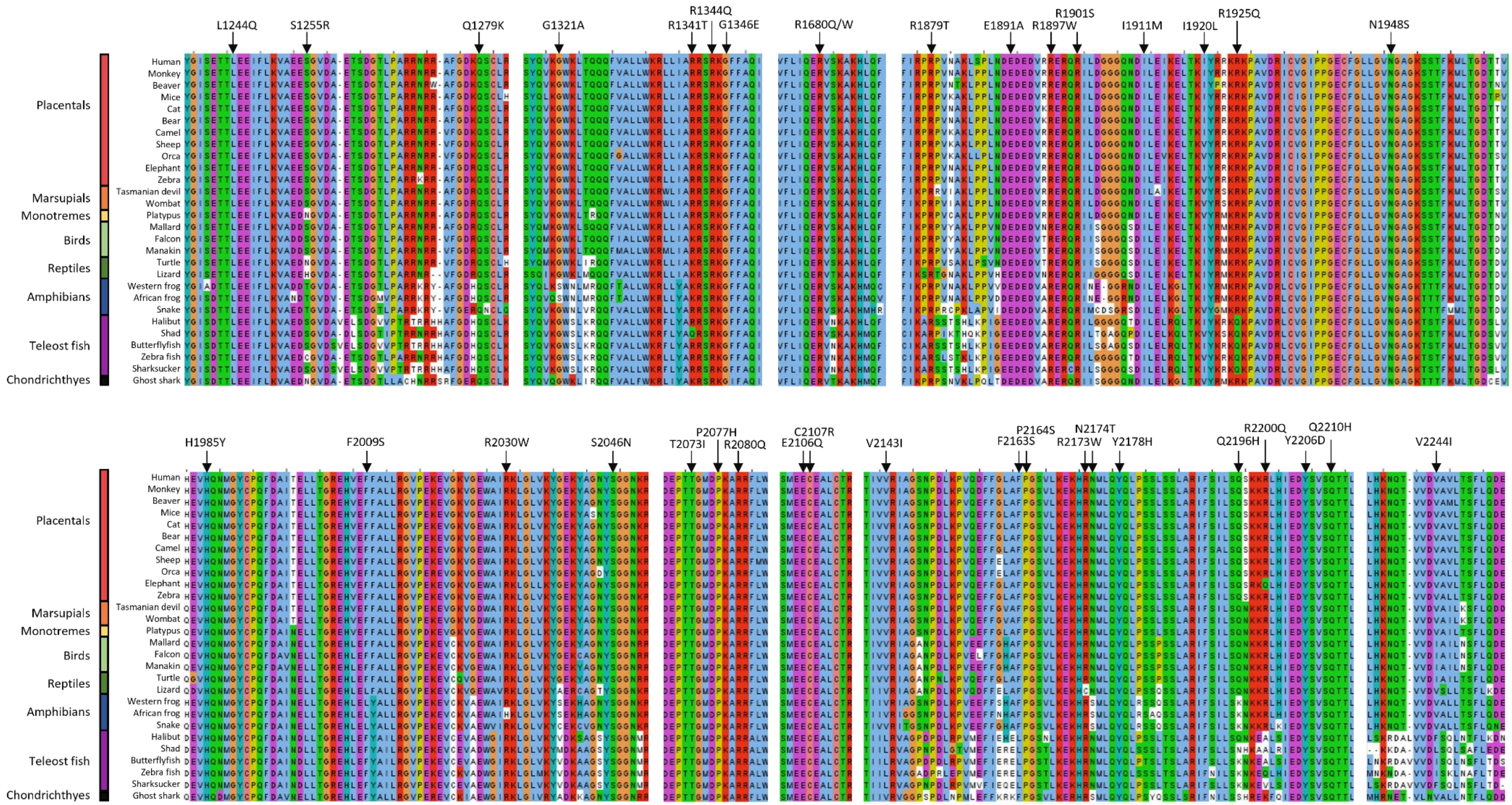
L1244Q
S1255R
Q1279K
G1321A
R1344Q
R1341T
G1346E
R1680Q/W
R1879T
E1891A
R1901S
R1897W
I1911M
I1920L
R1925Q
N1948S
Placentals
Marsupials
Monotremes
Birds
Reptiles
Amphibians
Teleost fish
Chondrichthyes
Human
Monkey
Beaver
Mice
Cat
Bear
Camel
Sheep
Orca
Elephant
Zebra
Tasmanian devil
Wombat
Platypus
Mallard
Falcon
Manakin
Turtle
Lizard
Western frog
African frog
Snake
Halibut
Shad
Butterflyfish
Zebra fish
Sharksucker
Ghost shark
H1985Y
F2009S
R2030W
S2046N
P2077H
T2073I
R2080Q
C2107R
E2106Q
V2143I
P2164S
F2163S
N2174T
R2173W
Y2178H
R2200Q
Q2196H
Q2210H
Y2206D
V2244I

Supplemental Figure S2: Multiple sequence alignment of 74 missense *ABCA1* variants. We sourced homologous protein sequences from the NCBI RefSeq database (76), employing standard BLAST sequence searches (77). Only sequences demonstrating a sequence identity exceeding 50% relative to human ABCA1 were selected for inclusion. The quality of these sequences was validated using the UniProt database (78). The alignments were executed in Jalview using the MUSCLE (28) algorithm and visualized employing the Clustal color scheme. The analysis encompassed a diverse range of species, including humans, *Saimiri boliviensis boliviensis* (Black-capped squirrel monkey), *Castor canadensis* (North American beaver), *Mus musculus* (House mouse), *Felis catus* (Cat), *Ursus arctos* (Brown bear), *Camelus bactrianus* (Bactrian camel), *Ovis aries* (Sheep), *Orcinus orca* (Orca or killer whale), *Elephas maximus indicus* (Indian elephant), *Sarcophilus harrisii* (Tasmanian devil), *Vombatus ursinus* (Common wombat), *Ornithorhynchus anatinus* (Platypus), *Anas platyrhynchos* (Mallard), *Falco naumanni* (Lesser kestrel), *Manacus candei* (White-collared manakin), *Mauremys reevesii* (Chinese pond turtle), *Lacerta agilis* (Sand lizard), *Xenopus tropicalis* (Western clawed frog), *Xenopus laevis* (African clawed frog), *Geotrypetes seraphini* (Gaboon caecilian), *Hippoglossus stenolepis* (Pacific halibut), *Alosa sapidissima* (American shad), *Chelmon rostratus* (Copperband butterflyfish), *Danio rerio* (Zebrafish), *Echeneis naucrates* (Live sharksucker), and *Callorhinchus milii* (Australian ghostshark).

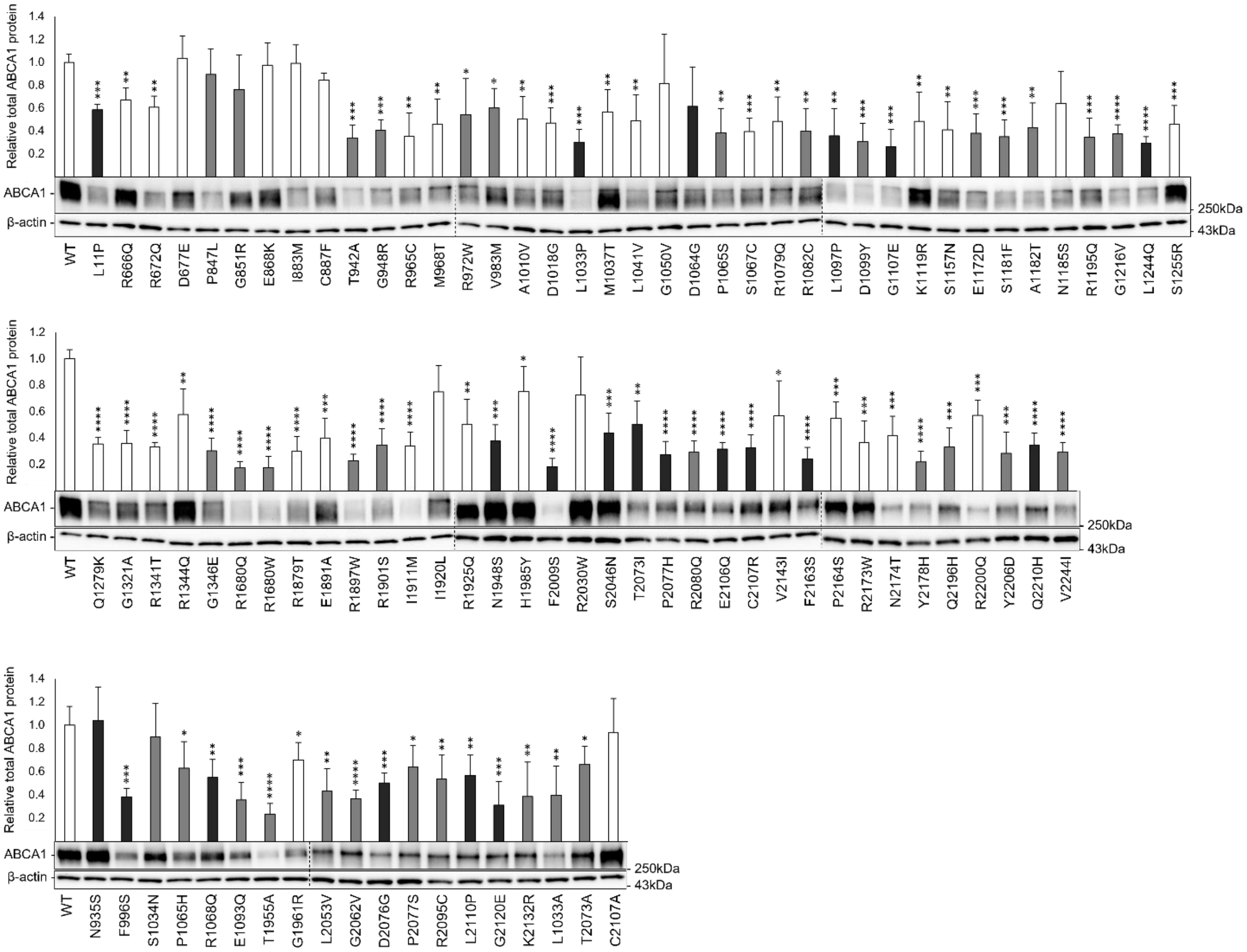

Supplemental Figure S3: Protein expression of *ABCA1* missense variants. Relative amounts of ABCA1 in lysates of HEK293 cells transiently transfected with *ABCA1* variants characterized as functionally normal (white columns), loss-of-function (black columns) or of uncertain significance (gray columns), corrected for loading control β-actin and normalized to WT ABCA1 (WT) in four independent experiments. Error bars represent 1 SD. *$P < 0.05$, **$P < 0.01$, ***$P < 0.001$, ****$P < 0.0001$, *t*-test versus WT ABCA1. One representative Western blot is displayed. Dotted lines designate merger of blots.

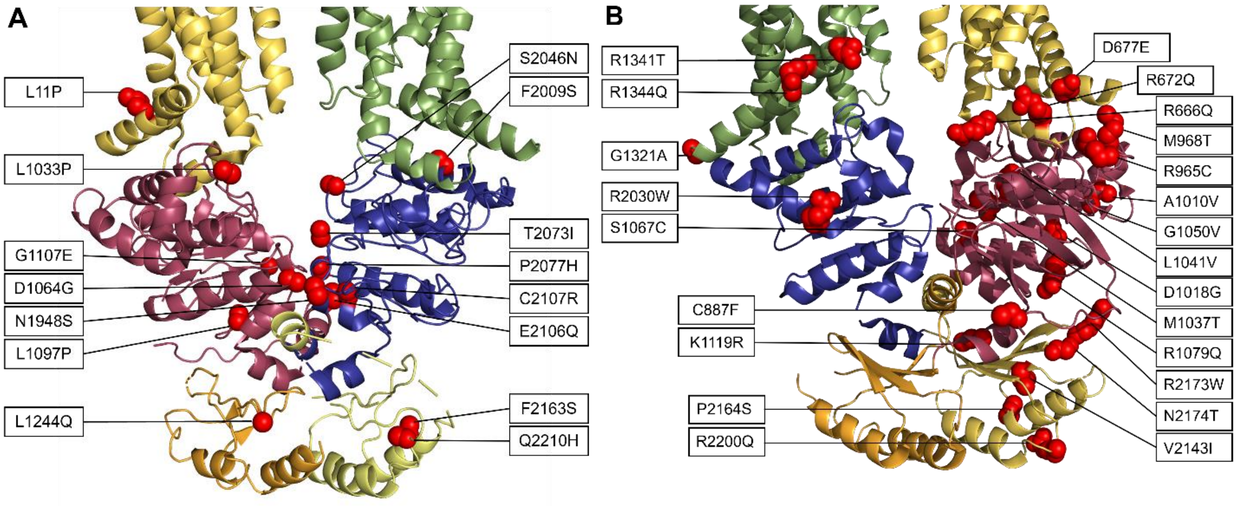

Supplemental Figure S4: Structural mapping of *ABCA1* Variants. The structural representations were generated using PyMOL (The PyMOL Molecular Graphics System, Version 3.1.6.1, Schrödinger, LLC, New York, NY). A: This figure depicts the localization of the 15 identified loss-of-function variants and is derived from Protein Data Bank entry 5XJY. Because p.L1244 is not depicted in the 5XJY model, the closest depicted residue (p.G1238) is marked. B: Localization of 23 out of the 35 functionally normal *ABCA1* variants analyzed in this study. The structural representation is based on Protein Data Bank entry 7ROQ. Because p.G1321 is not depicted in this model, the closest depicted residue (p.W1322) is marked. The remaining 12 variants are also not depicted in the 7ROQ protein model; most of these variants are also absent in other comparable ABCA1 protein models.

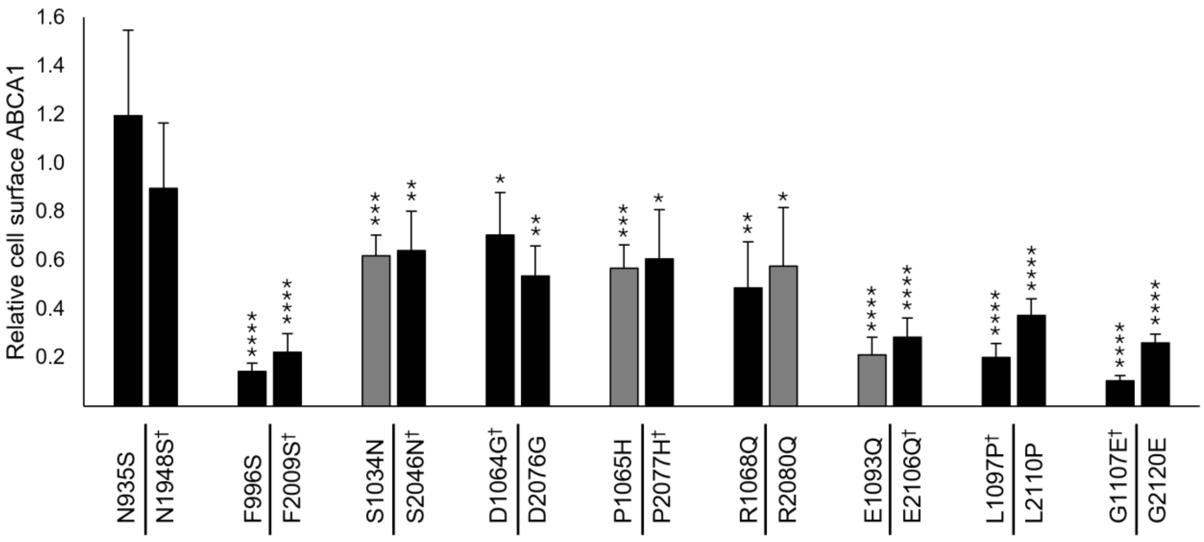

Supplemental Figure S5: Cell surface expression of equivalent *ABCA1* variant pairs. Relative amounts of cell surface ABCA1 were assessed for nine equivalent variant pairs of the two nucleotide binding domains (NBD1|NBD2), and normalized to WT ABCA1 in three independent experiments. Error bars represent 1 SD. **P* < 0.05, ***P* < 0.01, ****P* < 0.001, *****P* < 0.0001, *t*-test versus WT ABCA1. †Data already presented in Fig. 2 in main manuscript. Column colors correspond to functional characterization based on cholesterol efflux presented in Fig. 1 or Fig. 5B in main manuscript (black: loss-of-function, gray: variant of uncertain significance).

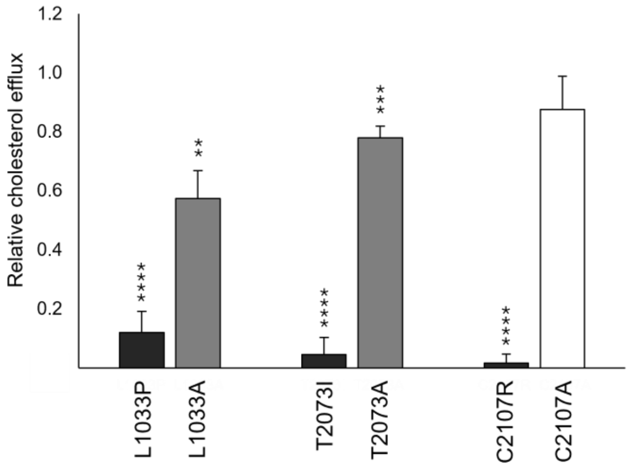

Supplemental Figure S6: Cholesterol efflux activity of alanine variants. Relative cholesterol efflux activity of alanine variants compared to loss-of-function variants in the same amino acid positions (black columns, data already presented in Fig. 1 in main manuscript), normalized to WT ABCA1 in four independent experiments. Variants were assessed to be functionally normal (efflux > 80% of WT, white columns) or of uncertain significance (efflux 41-80% of WT, gray columns). Error bars represent 1 SD. ***P* < 0.01, ****P* < 0.001, *****P* < 0.0001, *t*-test versus WT ABCA1.

Supplemental Table S3: Available clinical parameters for 13 of the *ABCA1* loss-of-function variants. Gender, zygosity and serum levels of total cholesterol (TC, mmol/l), HDL cholesterol (HDL-C, mmol/l) and ApoA1 (mg/dl) reported in subjects with loss-of-function variants in *ABCA1*. The additional *ABCA1* variant is indicated for the compound heterozygous subjects. Also included are values representing the normal range for HDL cholesterol and ApoA1, obtained from the Department of Medical Biochemistry, Oslo University Hospital, Norway.

Variant	Gender	Zygosity	TC	HDL-C	ApoA1	Reference
p.L11P	M	He	3.6[a]	0.39	62	(36)
p.L1033P	M	He	2.2[a]	0.47	82	(36)
p.D1064G	-	He	-	< 1.0	-	(56)
p.L1097P	M	Ho	1.5	0.05	0	(31)
p.L1244Q	-	He	5.3[b]	< 0.84	-	(67)
p.N1948S	-	He	-	0.62[c]	-	(71)
p.F2009S	M	c.He (p.D1099Y)	4.8	0.10	8	(33)
p.S2046N	F	c.He (p.P2077H)	2.0	0.05	< 1	(32)
p.T2073I	F	He	3.6[a]	0.41	73	(36)
p.P2077H	F	c.He (p.S2046N)	2.0	0.05	< 1	(32)
p.E2106Q	-	c.He (p.W590X)	-	< 0.1	< 1	Novel
p.F2163S	F	c.He (p.V2244I)	3.5[d]	0.74[d]	-	(72)
p.Q2210H	M	c.He (p.N1800H)	3.14	0.35	33	(74)
Normal range	M	-	-	0.8-2.1	100-200	-
	F	-	-	1.0-2.7	110-230	-

M: male; F: female; He: heterozygous; Ho: homozygous; c.He: compound heterozygous. [a] HDL-C + Non-HDL-C. [b] mean value of 56 study subjects. [c] mean value of six individuals of both genders in a family carrying the *ABCA1* variant. [d] mean values of two sisters both compound heterozygous for the *ABCA1* variants. Novel: identified at Unit for Cardiac and Cardiovascular Genetics, Oslo University Hospital.
